# Occult cardiac amyloidosis?

**DOI:** 10.18632/aging.102383

**Published:** 2019-10-19

**Authors:** Oscar Westin, Finn Gustafsson, Emil Fosbøl

**Affiliations:** 1 The Heart Center, University Hospital of Copenhagen, Rigshospitalet, Copenhagen, Denmark; 2 Department of Clinical Medicine, University of Copenhagen, Copenhagen, Denmark

**Keywords:** early diagnosis, occult cardiac amyloidosis, heart failure

Heart failure affects millions of patients worldwide and the impact on the health care systems is expected to increase as the population grows older. Approximately half of elderly patients with heart failure have preserved ejection fraction [1]. Heart failure with preserved ejection fraction is a heterogenous and poorly under-stood entity, spanning a broad spectrum of etiologies, of which cardiac amyloidosis is becoming increasingly recognized. Nonetheless, studies suggest that cardiac amyloidosis is markedly underdiagnosed:

- Autopsy studies of octogenarians have shown a 25% prevalence of myocardial transthyretin deposition [2]. 

- In a study of patients aged 60 years or older admitted for heart failure with preserved ejection fraction, and with left ventricular hypertrophy, a diagnosis of transthyretin cardiac amyloidosis was made in 13% after screening for the disease using scintigraphy [3]. 

- Studies on patients with severe aortic stenosis have shown a prevalence of cardiac amyloidosis in 6% of patients undergoing surgical valve replacement, and 16% of patients undergoing transcatheter valve replacement [4, 5], diagnosed by myocardial biopsy and scintigraphy respectively.

The underdiagnosing of cardiac amyloidosis is presumably due to the subtle clinical presentation in a population already burdened with comorbidities, and possibly due to the belief that diagnosis will not affect the outcomes, as no specific treatment has been available. Indeed, the prognosis in patients with cardiac amyloidosis is poor, but novel and specific pharmacological agents have recently been approved and shown to be effective [6, 7], warranting the quest for earlier diagnosis of cardiac amyloidosis. 

Due to the unaggressive diagnostic approach, the true epidemiology of cardiac amyloidosis is unknown. In clinical practice, certain signs are known to be found often in patients with cardiac amyloidosis, such as carpal tunnel syndrome and biceps tendon rupture. However, this has only been studied in small and highly selected patient series and the reverse – how often patients with carpal tunnel syndrome or biceps tendon rupture have or develop cardiac amyloidosis - has not been evaluated previously. Hence, it was unknown whether carpal tunnel syndrome is an independent risk marker of future cardiac amyloidosis. Clarifying this could prove valuable in early detection of the disease.

In a recent study, we utilized the Danish national registries to investigate the associations between previous surgery for carpal tunnel syndrome, cardiac amyloidosis, and adverse cardiovascular outcomes [8]. Comparing 56,032 patients treated surgically for carpal tunnel syndrome in the period 1996-2012, with age and gender matched controls from the general population, we found that carpal tunnel syndrome was associated with a 12-fold higher risk of diagnosed cardiac amyloidosis compared to control subjects, but the absolute incidence risk of incident cardiac amyloidosis was low. Carpal tunnel syndrome was also associated with a significantly increased risk of other adverse cardio-vascular events such as heart failure, atrial fibrillation, atrioventricular block and pacemaker/ICD implantation.

Being an observational study the assessment of causality is limited. Nonetheless, our results suggest that carpal tunnel syndrome may be an early marker for risk of developing cardiac amyloidosis and adverse cardiovascular events, and thereby provide an opportunity for early disease detection. Given the low absolute risk of developing (diagnosed) cardiac amyloidosis more studies are needed to determine which patients with carpal tunnel syndrome should undergo further testing. As the diagnosis often can be made safely and non-invasively using a combination of biochemistry, echocardiography and scintigraphy, the threshold for investigating appropriate patients might be lowered allowing for screening of individuals at risk.

Previously considered a rare disease of limited clinical interest with no treatment available, increasing evidence is pointing towards cardiac amyloidosis being a more common condition for which effective treatment is emerging. As treatment for cardiac amyloidosis appears to be more effective if initiated before manifest heart failure (at which time the patient is normally referred to cardiac evaluation), improved insight in the true epidemiology of cardiac amyloidosis is crucial to enable early identification of patients suitable for screening.

Although it will need refining, our study suggests one potential pathway for early disease detection and consequently better treatment of cardiac amyloidosis. Future studies will determine the clinical characteristics which should mandate work-up for cardiac amyloidosis. This, together with more information on optimal use of the new disease modifying drugs, will finally improve the quality of life and survival of patients with cardiac amyloidosis.
